# Systemic lupus erythematosus in Saudi children: Long-term outcome

**DOI:** 10.1186/1546-0096-9-S1-P257

**Published:** 2011-09-14

**Authors:** Sulaiman M Al-Mayouf

**Affiliations:** 1 Department of Pediatrics, Section of Rheumatology, King Faisal Specialist Hospital and Research Center, Riyadh, Saudi Arabia

## Objective

To report the long-term outcome of a cohort of Saudi children with systemic lupus erythematosus (SLE)

## Methods

Medical records of all children with SLE treated between 1990 and 2010 at King Faisal Specialist Hospital and Research Center, Riyadh were retrospectively reviewed. The long-term outcome measured by Systemic Lupus International Collaborating Clinics American College of Rheumatology Damage Index (SDI) and death related to SLE. The collected data included: gender, age at disease onset, follow up duration and clinical features at diagnosis and last follow up visit as well as treatment.

## Results

One hundred and forty two patients (108 girls and 34 boys) were included. The mean age at onset of SLE was 84.5 months (18- 144 months) while the mean age at diagnosis was 111.6 months (24- 156 months) and the mean duration of follow up was 62 months (8- 235 months). All patients treated with corticosteroid and immunosuppressive drugs; 93 (69.3%) patients received cyclophosphamide and 18 patients treated with rituximab. Overall, 66 (46.5%) patients had damage with a mean SDI score of 2.6 within a mean of 62 month of follow up duration. Damage accrual was mostly in the growth (26.8%), renal (16.9%) and neuropsychiatric (14%) domains. 11 patients had progressive renal disease and required dialysis, 4 of them underwent renal transplant. There were 11 deaths related to SLE; 8 of them due to sepsis. Logistic regression analysis showed renal disease required dialysis and renal transplant associated significantly with male gender independently of age of disease onset. In contrast death related to SLE influenced by early onset disease.

## Conclusion

Our cohort indicates that the long-term outcome was satisfactory. Male gender and early onset disease probably influence the long-term outcome of SLE in children. Infection remains an important cause of death in children with SLE.

